# Dynamic Hyaluronan drives liver endothelial cells towards angiogenesis

**DOI:** 10.1186/s12885-018-4532-1

**Published:** 2018-06-11

**Authors:** Sampa Ghose, Subhrajit Biswas, Kasturi Datta, Rakesh K. Tyagi

**Affiliations:** 1 Department of Medical Oncology, All India Institute of Medical Sciences, Ansari Nagar, New Delhi, 110029 India; 2 Special Centre for Molecular Medicine, Jawaharlal Nehru University, New Delhi, India; 3 Amity Institute of Molecular Medicine and Stem Cell Research, Amity University Uttar Pradesh (AUUP), Sector 125, NOIDA, Uttar Pradesh, 201313 India; 4 School of Environmental Sciences, Jawaharlal Nehru University, New Delhi, India

**Keywords:** Hyaluronic acid or Hyaluronan, Liver endothelial cells, Angiogenesis

## Abstract

**Background:**

Angiogenesis, the formation of new blood vessels from pre-existing vasculature is essential in a number of physiological processes such as embryonic development, wound healing as well as pathological conditions like, tumor growth and metastasis. Hyaluronic acid (HA), a high molecular weight polysaccharide, major component of extracellular matrix is known to associate with malignant phenotypes in melanomas and various other carcinomas. Hyaluronic acid binding protein 1 (HABP1) has been previously reported to trigger enhanced cellular proliferation in human liver cancer cells upon its over-expression. In the present study, we have identified the HA mediated cellular behaviour of liver endothelial cells during angiogenesis.

**Methods:**

Endothelial cells have been isolated from perfused liver of mice. Cell proliferation was studied using microwell plates with tetrazole dye. Cell migration was evaluated by measuring endothelial monolayer wound repair as well as through transwell migration assay. Alterations in proteins and mRNA expression were estimated by immunobloting and quantitative real time PCR using Applied Biosystems. The paraformaldehyde fixed endothelial cells were used for immuno- florescence staining and F-actin detection with conjugated antibodies. The images were captured by using Olympus florescence microscope (IX71).

**Results:**

We observed that administration of HA enhanced cell proliferation, adhesion, tubular sprout formation as well as migration of liver endothelial cells (ECs). The effect of HA in the rearrangement of the actins confirmed HA -mediated cytoskeleton re-organization and cell migration. Further, we confirmed enhanced expression of angiogenic factors like VEGF-A and VEGFR1 in endothelial cells upon HA treatment. HA supplementation led to elevated expression of HABP1 in murine endothelial cells. It was interesting to note that, although protein levels of β- catenin remained unaltered, but translocation of this protein from membrane to nucleus was observed upon HA treatment, suggesting its role not only in vessel formation but also its involvement in angiogenesis signalling.

**Conclusions:**

The elucidation of molecular mechanism (s) responsible for HA mediated regulation of endothelial cells and angiogenesis contributes not only to our understanding the mechanism of disease progression but also offer new avenues for therapeutic intervention.

**Electronic supplementary material:**

The online version of this article (10.1186/s12885-018-4532-1) contains supplementary material, which is available to authorized users.

## Background

The metastatic spread of tumor cells is the most lethal aspect of cancer, which often occurs through enhanced vascularization. The initiation of angiogenesis embarks with the local release of pro- and anti-angiogenic growth factors by endothelial cells (ECs). Such release occurs in response to disease- or injury- induced inflammation. Vascular endothelial growth factors (VEGF) and glycosaminoglycans are important regulators [1]. The VEGF- induced cytoskeleton reorganization also plays a crucial role in the angiogenic processes [2, 3], though the intracellular signals leading to these events are not yet clear. A series of sequential events are involved in angiogenesis including local degradation of endothelial basement membrane by the action of proteases, formation of a lumen, proliferation and migration of endothelial cells that gives rise to a functional vessel.

The chief component of the extracellular matrix (ECM), hyaluronan or hyaluronic acid (HA) promotes tumor growth by providing a loose matrix for cancer cells to migrate and adhere [4]. Since long time, the polysaccharide HA, has been used in a wide variety of medical fields as diverse as neurosurgery to cutaneous wound healing. HA is comprised of 10,000 repeating units of (β,1→4)-D glucuronic acid-(β,1→3)-N-acetyl-D-glucosamine [5], and its molecular weight ranges from 400 Da to several MDa. Within the ECM the degraded fragments of HA, termed as low molecular weight hyaluronan (LMW-HA), have been reported as significant regulator of angiogenesis [6]. HA has also received great attention due to multi-functional regulation on related biological functions such as inflammation, wound healing and tumor growth [7]. Previously we identified the hyaladherin, the hyaluronic acid binding protein 1 (HABP1), from rat liver using HA-affinity column chromatography [8]. We have demonstrated for the first time, that *Plasmodium falciparum* infected RBCs use HABP1 as a receptor to bind to human endothelial cells [9]. Our studies have shown that overexpression of HABP1 in the human liver cell line HepG2 (HepR21) induces high endogenous glutathione level and enhanced cellular proliferation along with increased endogenous level of HA and intercellular HA cables [10] whereas HABP1 overexpression leads to ROS-mediated apoptosis in normal fibroblasts [11, 12]. The elevated level of HA is associated with hyper-proliferative and invasive tumorigenesis [13, 14].

Several studies are emphasizing the involvement of HA in endothelial cell proliferation, migration and new vessel formation [15]. However, very few reports are available on the effect of HA on liver sinusoidal endothelium. In the liver, HA is synthesized mostly by the sinusoidal pericyte and the hepatic stellate cells (HSCs); while it is degraded by the liver sinusoidal endothelial cells (LSECs) [16]. The role of HABP1 in cell-adhesion is well established and in combination with HA, it facilitates the process of adhesion and de-adhesion during mitotic stages [10]. The another major adhesion molecule, β-catenin is not only one of the key molecules regulating the hepatic zonation pattern [17] but also acts as transcriptional co-regulator and an adaptor protein for cellular adhesion. Postnatal liver growth and development is also dependent on β-catenin activity. Extensive cell proliferation occurs in the liver after birth, in conjunction with a substantial increase in β-catenin protein and its nuclear translocation [18]. In fact liver metastasis is often supported by abnormal β-catenin expression and localization [19]. β-catenin accumulation within the nucleus or cytoplasm has been found remarkably in more than half of all cancers and is related to increased tumorigenicity [20]. The biological events that couple HA and β-catenin function to angiogenesis are still unknown.

The present study has focused on identification of HA mediated cellular behaviour of liver endothelial cells involving β-catenin activation and its influence on angiogenic signals for cellular adhesion and wound healing. We have worked on how HA stimulates endothelial cell migration and adhesion through VEGF, leading towards angiogenesis in vitro. The cellular roles of HA are perpetrated through molecular interactions with HA-binding proteins or hyaladherins. In particular, we have demonstrated here the role of the VEGF receptors involved in initiating the coordinated signals that leads to actin based motility and angiogenesis.

## Methods

### Endothelial cell isolation and cell culture

A reproducible method has been used to isolate endothelial cells (ECs) from murine liver as described earlier [21] with modifications. After sacrificing the mice, liver was perfused with warm PBS by injecting needle to flush out blood. The perfused liver was then put into serum-free media with antibiotics and minced into small pieces. Minced liver was incubated in 7–8 ml collagenase (500μg/ml) for 15 min. After spinning down at 2000 rpm for 5 min, supernatant was removed and the collagenase treatment was repeated for 2–3 times for total digestion of big chunks of tissues. Completely digested cells were re-suspended in complete media with 20% FBS, passed through 40 μm filter and plated on a 0.1% gelatin coated plate. The cells were incubated for 3–4 h. Media was splashed directly into cells for a couple of rounds and washed extensively with serum-free media to wash off all other cell types except ECs. ECs were cultured on collagen-coated plates and grown in MCDB 131 (HiMedia) media supplemented with 20% FBS, 100 μg/ml penicillin and 100 μg/ml streptomycin (complete medium). The cultures were maintained in a humidified incubator maintained at 5% CO_2_ and 95% air atmosphere at 37 °C. EC phenotype was confirmed by immuno-cytochemistry with anti- CD31 (Abcam) and anti-VEGF antibody (Thermo Fisher Scientific).

### Cell proliferation assay / MTT assay

The low molecular weight hyaluronic acid from umbilical cord (LMW-HA) were purchased from Sigma-Aldrich and used to study its effect on ECs with final concentration of 10 μg/ml [22]. ECs were plated at a density of 2 × 10^4^ cells/ well in 48-microwell plates and were treated with HA (10μg/ml) for 24–72 h. At the indicated time points, cells were treated with 10 μl yellow tetrazole dye [3-(4,5-Dimethylthiazol-2-yl)-2,5- diphenyl tetrazolium bromide], MTT(1 mg/ml; in PBS) for 4 h at 37 °C in a dark, humidified, 5% CO_2_ environment as per instructions from HiMedia (#TC191) and our previously standardized protocol [23]. The precipitate formed was solubilised in 150 μl isopropanol after discarding the media and then absorbance was recorded at 570 nm using a plate reader.

### Adhesion assay

For both untreated and HA treated (10μg/ml, for 48 h) ECs, 1 × 10^5^ cells were seeded in each well of a 48 well plate. Non-adherent cells were removed after 2 and 4 h by washing with PBS. Only adherent cells were fixed by treating with 1% glutaraldehyde for 10 min and stained with 0.1% (*w*/*v*) crystal violet (Sigma-Aldrich) for 25 min. The cells were washed and solubilized in 1% TritonX100 (Sigma-Aldrich) for overnight and the absorbance was measured at 620 nm. Samples were taken in triplicates for each time point.

### Wound healing assay

Endothelial migration was evaluated by measuring endothelial monolayer wound repair. The confluent monolayers of cells were scratched identically with the narrow edge of a 200μl tip. The monolayer was washed with serum-free media and treated with HA (10 μg/ml). ECs were observed at different time point to see the extent of wound healing. Some wells were kept untreated as control. DIC images were captured at the time of scratching and up to 48 h under an inverted phase contrast microscope.

### Tubule formation assay

Vascular tubule formation assay was performed in a three-dimensional culture on top of growth factor reduced matrigel (Becton Dickinson). After the treatment, cells were trypsinized and seeded on solidified matrigel in 48 well plates. The vascular sprout or growing tubule structure was photographed from randomly selected fields after 18 and 42 h of HA treatment under the microscope. Untreated endothelial cells were used as control. Tubules were quantified per field for statistical validation.

### Migration assay

Cell migration assay was performed using transwell plate (HiMedia). HA treated and untreated ECs were trypsinized and re-suspended in serum free MCDB131 media (HiMedia). 1X10^5^ cells were added in the upper chamber of transwells (8.0 μm). 500 μl of MCDB131 media with 20% FBS (HiMedia) was added to the lower chamber of each well which act as a chemoattractant. The plate was covered and incubated at 37^ 0^C in 5% CO_2_ incubator for at least 24 h. The cells on the lower surface of transwell membrane were fixed with 2% paraformaldehyde for 10 min followed by staining with 0.1% Crystal violet for 1 h. The number of migrated cells were counted in five different fields under the microscope and represented in a histogram showing the average number of migrated cells through the porous transwell membrane.

### Immunoblot analysis

Untreated and HA treated (24 and 48 h) ECs were lysed in RIPA buffer. Equal amounts of protein samples were electrophoresed by 10% SDS-PAGE. Following transfer into the PVDF membrane, proteins were immuno detected by probing the blot with specific antibodies. The bound antibody complexes were detected using enhanced chemi-luminescence (ECL) system.

### RNA isolation and qPCR

Total RNA was isolated from untreated and HA treated endothelial cells (10 μg/ml for 24 and 48 h) using TRI reagent according to the manufacturer’s instructions. RNA was quantitated and 5 μg of total RNA was used for cDNA synthesis with the help of RevertAid H Minus First cDNA Synthesis Kit with random hexamers as primers (Thermo Scientific #K1631). GAPDH, β-catenin, VEGF and VEGFR1 were amplified by PCR using 1 μl of cDNA. Amplification of GAPDH gene was taken as a positive control. The PCR products were resolved on 1.5% agarose gel and visualized by ethidium bromide staining. qPCR was performed using the Eva Green PCR Master Mix (G-biosciences, cat #786–858) and ABI Prism 7500 (Applied Biosystems). Amplification was done by denaturation for 10 min at 95 °C followed by 40 cycles of 15 s at 95 °C, 30s at 60 °C and 30 s at 72 °C. Relative quantitation was done by parentizing threshold cycle (*Ct*) values of each sample gene with *Ct* values of *GAPDH*. *ΔCt* corresponds to the difference between the *Ct* of the genes of interest and the *Ct* of *GAPDH* [10]. Data are presented as fold changes of mRNAs, which were calculated for HA treated endothelial cells (derived from 2^*-ΔΔct*^ method) in comparison with untreated control samples. The primers were designed from NCBI (https://www.ncbi.nlm.nih.gov/tools/primer-blast/) and sequences are mentioned in Additional file 1: Table S1.

### Immuno-florescence staining and F-actin detection

Endothelial cells were seeded on 0.1% gelatin coated cover slip and incubated for 4 to 5 h for attachment. Once the cells get attached, they were treated with 10μg/ml HA. After 48 h of HA treatment, cells were fixed in 4% (*v*/v) paraformaldehyde in PBS for 20 min at room temperature. Cells were permeabilized using 0.1% TritonX-100 in PBS (v/v) for 5 min at room temperature and the excess detergent was washed off with PBS. Cells were blocked with 5% BSA/PBS for 1 h at room temperature followed by incubation with primary antibody (1:1000) in 1% BSA/PBS for overnight at 4 °C. Cells were washed thrice with PBS and then incubated with Cy3 tagged secondary antibody (Sigma) for 1 h. Hoechst 33342 (Sigma) was co-incubated with the secondary antibody to visualize the nucleus. Cells were again washed with PBS thrice, 5 min each and mounted on 30% glycerol in PBS. To see actin organization, paraformaldehyde fixed cells were permeabilized and incubated with fluorophore-conjugated phalloidin along with hoechst for 1 h. After incubation the cells were rinsed with PBS several times, mounted on 30% glycerol and images were captured in a florescence microscope (Olympus IX71).

### Statistical analysis

All data are presented as mean ± S.D. (standard deviation) from at least three separate experiments. Student’s *t* test was applied to evaluate the differences between treated and control groups. Data from multiple groups were analysed by one-way ANOVA. For all the tests, the level of significance was kept at *p* < 0.05.

## Results

### Exogenous HA treatment induces proliferation of endothelial cells

**Fig. 1:**
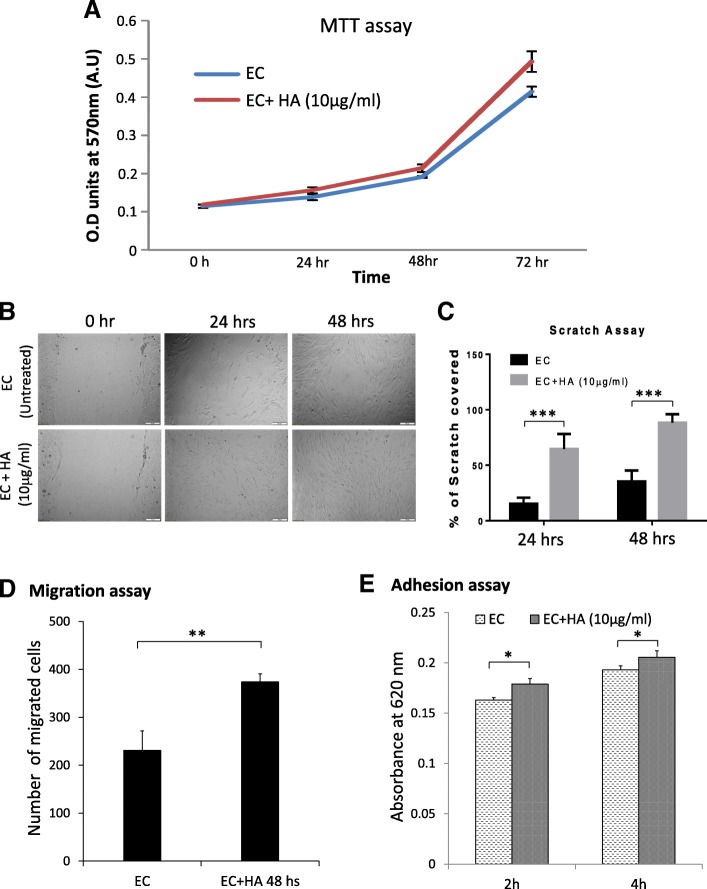
HA Influence the proliferation, wound healing, migration and adhesion of endothelial cells. **a** Cell survivability assay (MTT) was performed for untreated and HA-treated (10μg/ml) ECs to examine the effect of HA on proliferation rate. Three wells for each condition were taken for different time points. Same number of cells were seeded in triplicates for each time point along with a set of untreated cells as controls **b** Images of scratch assay showing the influence of HA on ECs migration to recover the wound. Scale bar, 100 μm. **c** Percentage of scratch covered after HA treatment were quantified after 24 and 48 h **d** Graph showing the number of migrated cells through the porous transwell membrane in untreated control and HA treated (10μg/ml for 48 h) ECs. **e** Graph showing change of adhesion property of cells with HA treatment (10μg/ml for 48 h) after 2 and 4 h in comparison to equal number of untreated control ECs. *p < 0.05; **p < 0.01, ***p < 0.001

Within the liver, HA is mostly synthesized by the sinusoidal pericytes, the hepatic stellate cells (HSCs) [24, 25] and removed from circulation through degradation especially by liver sinusoidal endothelial cells (LSEC) [26, 27]. Although HA can also be metabolized in vitro by cultured hepatocytes and Kupffer cells [28, 29], the role of these cells in the physiological HA metabolism in vivo was shown as minor importance in literature compared to LSEC. Here we explored the effect of exogenous HA on proliferation of liver ECs isolated from mice. We treated ECs with 10μg/ml HA upto 72 h and performed MTT assay at different mentioned time points. A positive impact of HA supplementation has been observed on EC proliferation (Fig. 1a). In fact ECs, treated with HA have significantly higher growth rate than untreated ECs, suggesting the probable constructive role of HA in liver microenvironment.

### Faster wound healing and improved cellular adhesion due to HA treatment

The angiogenic response during chronic liver injury and cirrhosis are associated with formation of a dense neovasculature fibrotic septa surrounding regenerative nodules. In hepatocellular carcinoma (HCC), the intra-tumorous angiogenesis is accompanied by peri-tumorous angiogenesis in the adjacent liver tissue [30, 31]. HA stimulate angiogenesis and become extraordinarily active in pathological events [32]. These pathologic vessels become capillarized, defenestrated and form a more classic vascular basement membrane [33]. Liver endothelial cells in these circumstances take on an “activated” angiogenic phenotype which includes altered surface markers [34, 35] and changes in both morphology and behaviour [35], allowing not only increased proliferation but also invasion, adhesion and wound healing. Similar changes can be seen in the settings of portal hypertension [36], HCC [37] and during the aging process [38].

Here we assumed that there would be an association of HA uptake with endothelial cell migration and adhesion, two key events associated with angiogenesis. To elucidate the effects of HA on wound healing scratch assay was performed. The result showed that, higher number of HA treated endothelial cells migrated to the wounded area, leading to faster recovery of wound in comparison to the untreated cells (Fig. 1b & c). Further, the result of migration assay performed using transwell membrane also corroborates the findings of wound healing assay (Fig. 1d). Therefore, both the date suggested that, cellular migration of ECs in vitro culture is significantly influenced by HA treatment. In addition, the effect of HA on cellular adhesion has also been verified. Adhesion assay for ECs was performed in presence or absence of HA in culture media. Interestingly, increased adhesion has been observed in HA treated ECs in comparison to untreated cells (Fig. 1e).

### Enhanced tubule formation in liver endothelial cells upon HA treatment

**Fig. 2:**
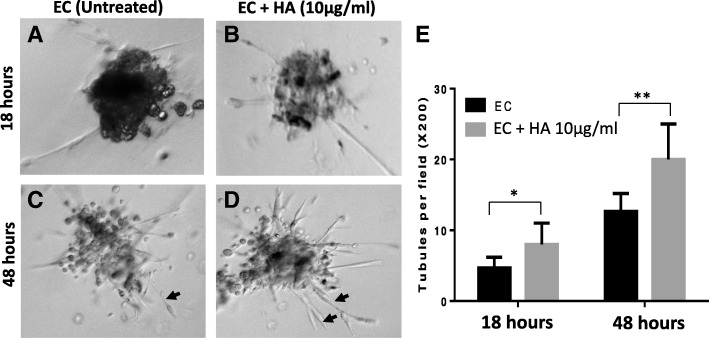
Effect of HA treatment on tubule formation of ECs. The influence of HA on vascular tubule formation of ECs are shown with DIC images of Matrigel assay. Murine ECs with HA treatment (right panel) were found with more growing vascular sprouts in comparison to untreated ECs (left panel) after 18 h (**a**, **b**) and 48 h (**c**, **d**) respectively. **e** The quantification of tubules (indicated with arrows) were measured per microscopic field after indicated time points. *p < 0.05; **p < 0.01

Tumor induced angiogenesis requires migration and remodelling of endothelial cells derived from pre-existing blood vessels. Vascular endothelial growth factor (VEGF) is the most implicated growth factor in the development of neo-vessels. With a goal to explore whether HA treatment has any effect on tubule formation, we assessed the vascular network formation in a three-dimensional matrigel assay. In this assay, ECs were added on solidified matrigel and monitored for growth of tubules. The DIC pictures were taken for both HA treated and untreated ECs growing on matrigel at different time points. More number of vascular sprouts were appeared in case of the HA treated ECs as compared to untreated cells (Fig. 2a). Result was confirmed by quantifying the tubules per microscopic field (Fig. 2b), implicating a direct role of HA on vessel formation.

### Elevated expression of pro-angiogenic factors in endothelial cells upon HA treatment

Liver sinusoidal endothelial cells (LSECs) act as a filter between the lumen of the hepatic sinusoids and the surrounding hepatocytes. These sinusoidal endothelial fenestrations are altered by various liver diseases, diabetes mellitus and old age due to the influence of cytokines and hormones [39]. The various agents that influence the number and diameter of fenestrations are calcium, serotonin, ethanol, prostaglandins, endothelin-1 etc. [40, 41]. But recent reports imply an immense influence of vascular endothelial growth factor (VEGF) on genesis of fenestrations [42]. During this process hepatocytes exhibit molecular heterogeneity based on their location within the hepatic lobule or hepatic zonation [43]. β-catenin is one of the key molecules regulating the zonation pattern. It is required for expansion of hepatoblasts during early stages of hepatic morphogenesis and for proper specification of hepatoblasts as well as hepatocyte maturation during later stages. β-catenin regulates the expression of genes such as glutamine synthetase (GS) and certain cytochrome P450 enzymes (CYPs), such as Cyp1a2 and Cyp2e1 [44]. In addition, β-catenin signalling is also essential for the initiation of liver regeneration (LR).

**Fig. 3:**
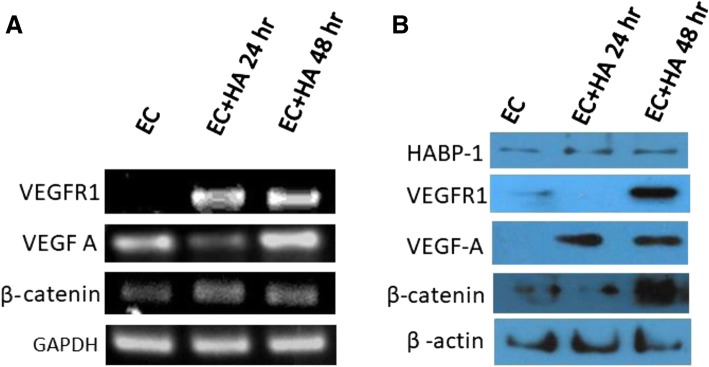
HA treatment up-regulates pro angiogenic factors within ECs. **a** Reverse transcriptase PCR with VEGFR1, VEGFA and β- catenin primers confirmed increased mRNA level in murine ECs upon HA treatment at mentioned time points. **b** Immunoblot with anti-HABP1, anti- VEGFR1, anti- VEGFA and anti-β-catenin antibody showed increased VEGFR1, VEGFA and β- catenin protein levels after HA treatment of 24 and 48 h. Equal protein loading is confirmed by probing the blot with β-actin antibody

**Fig. 4:**
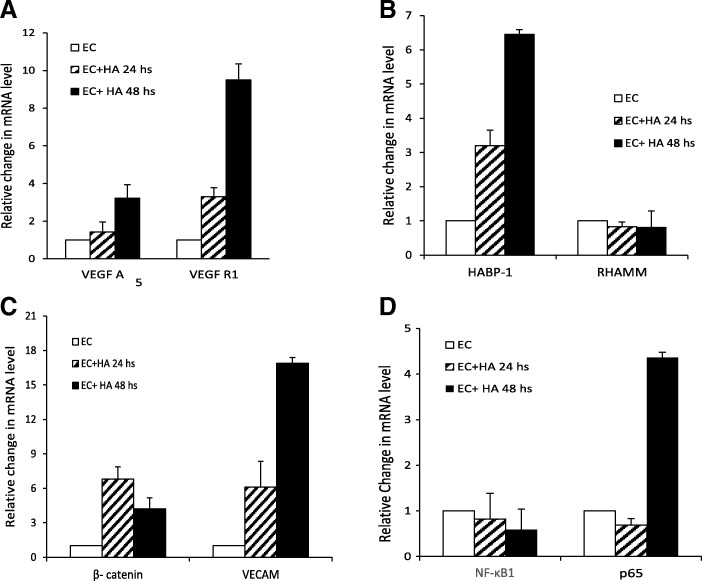
Alteration in mRNA expression of angiogenic factors in ECs upon HA treatment. Real-time PCR showing the relative fold change in mRNA level of several pro-angiogenic factors like **a** VEGF (Vascular endothelial growth factor) and VEGFR1 (Vascular endothelial growth factor receptor 1), **b** HABP1 (Hyaluronic acid binding protein1) and RHAMM (Hyaluronan mediated motility receptor) **c** β-catenin (Catenin β1) and VECAM (Vascular cell adhesion molecule) **d** NF-κB1 (p50 subunit of nuclear factor kappa-light-chain-enhancer of activated B cells) and p65 (nuclear factor NF-kappa-B p65 subunit)

To understand whether HA mediates angiogenesis by up-regulation of these key angiogenic factors, we subsequently estimated both the mRNA as well as protein levels of VEGF-A, VEGFR1 and β-catenin in whole cell lysates of HA treated and untreated ECs. Elevated mRNA levels of typical angiogenic growth factors like VEGFR1, VEGF-A, β-catenin (Fig. 3a) have been observed in ECs upon HA treatment. Immunoblotting with β-catenin, VEGF-A and VEGFR1 antibodies also elucidated augmented protein expression of VEGF-A, VEGFR1, β-catenin and a little elevation in HABP1 level in HA treated ECs as compared to the untreated ECs (Fig. 3b). Quantitative PCR (qPCR) analysis for mRNA expression level of crucial regulators of angiogenic pathway demonstrated a significant up-regulation of VEGFA (~ 3.2 fold) and a huge increase in VEGFR1 mRNA (~ 9.5 fold) level (Fig. 4a). A relative increase in the mRNA level of HABP1 (~ 6.5 fold) and β-catenin (~ 4.2 fold) has also been observed in the HA treated ECs. Among the three principal cell surface receptors for HA, only VECAM (Vascular Endothelial Cell Adhesion Molecule) mRNA was augmented in HA treated ECs (after 48 h of treatment) as compared to the untreated ECs. But, no significant change was observed for mRNA levels of RHAMM (Receptor for Hyaluronan Mediated Motility) (Fig. 4b-c). Although no change in the overall mRNA level of NF-κB1 (p50 subunit of NF-κB) has been observed upon HA treatment but a significant increase in transcription factor p65 (nuclear factor NF-kB, p65 subunit) which is very crucial for nuclear translocation and NF-κB signalling (Fig. 4d).

### Alterations in F-actin distribution and differential localization of HABP1 and β-catenin upon HA treatment in ECs

**Fig. 5:**
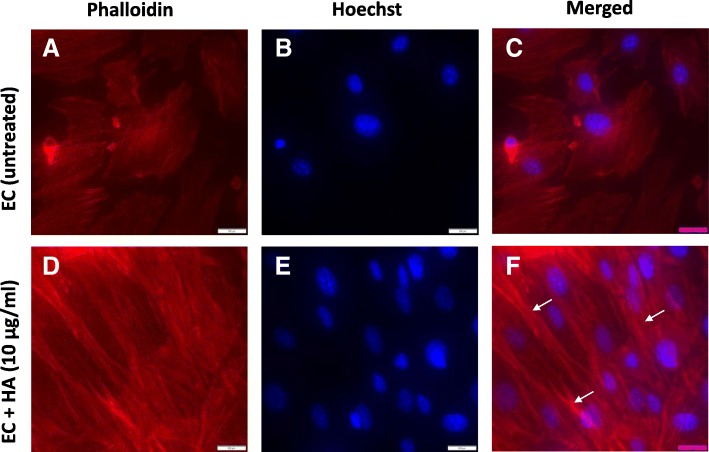
HA induces morphological and cytoskeletal alterations in ECs. **a**, **d** Cells were stained with Phalloidin to detect F-actin, (**b**, **e**) stained with Hoechst and (**c**, **f**) showing merged picture of phalloidin and Hoechst of untreated ECs (upper panel) vs HA treated (10μg/ml) ECs for 48 h (lower panel). The higher fluorescence intensity of phalloidin in ECs were indicated with arrows. Scale bar, 20 μm

**Fig. 6:**
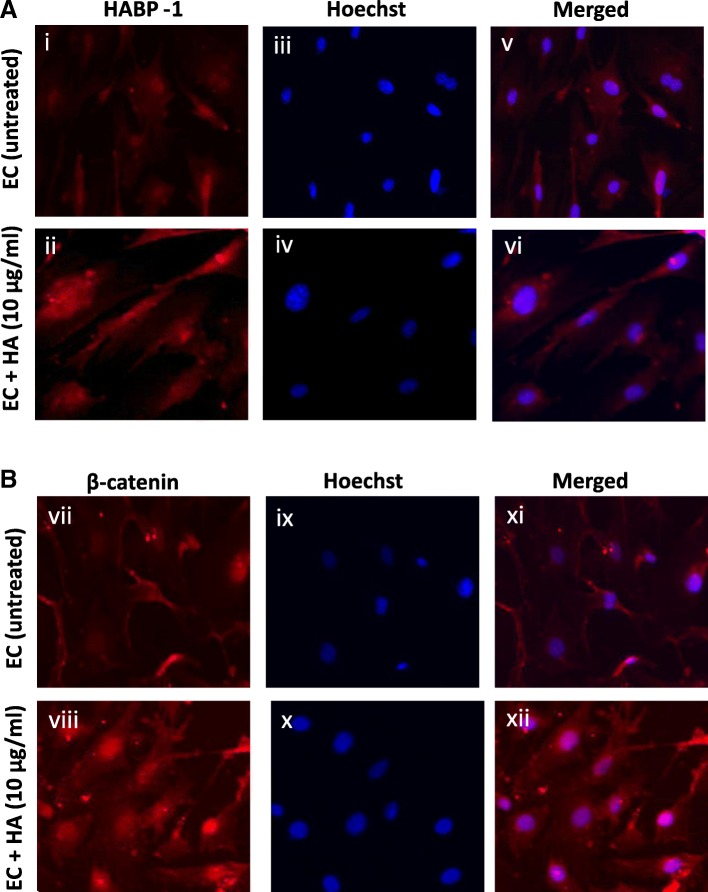
HA treatment influences localization of HABP1 and β-catenin in ECs. **a** Immunofluorescence staining showed cellular localization of endogenous HABP1 in mouse liver ECs, without HA treatment and with HA treatment. (i, ii) showing localization of HABP1, (iii, iv) staining with hoechst for detecting nucleus and (v, vi) merged picture of HABP1 and hoechst. **b** Cellular localization of endogenous β-catenin in mouse liver ECs, without HA treatment and with HA treatment. (vii, viii) showing localization of β-catenin, (ix, x) staining with hoechst for detecting nucleus and (xi, xii) merged picture of β-catenin and hoechst

Apart from its role in angiogenesis, VEGF is also required for a tight regulation of the contractile and non-contractile states of actin cytoskeletal organization. It is also believed that the orientation of the cytoskeleton inside the cells can control the orientation of the matrix produced outside. The cytoskeletal integrity of the HA treated and untreated ECs was studied here by observing the expression profile of F-actin. The actin network (red) were labelled with fluorophore-conjugated phalloidin (Fig. 5a & d) and the nuclei (blue) were labelled with Hoechst 33342 (Fig. 5b & e). Rhodamine-phalloidin staining indicated significantly altered distribution of F-actin in the HA treated ECs as compared to control untreated ECs (Fig. 5c). Increased fluorescence intensity of phalloidin (Fig. 5d & f) indicates that the exposure of murine sinusoidal ECs with HA promotes polymerization and reorganization of F-actin. Previous studies have revealed dynamic subcellular localization of the hyaladherin, HABP1 [45]. Thus, presuming a role of HA supplementation on the sub-cellular localization pattern of HABP1 in the ECs, immunofluorescence studies were performed using anti-HABP1 antibody in both the treated and untreated ECs. Fluorescence microscopy revealed mostly nuclear or perinuclear localization of HABP1 in the untreated ECs, while HA treated ECs showed a higher expression of HABP1 though out the cells, indicating a possible translocation of HABP1 from the nuclear periphery to the surface. Moreover, the immunofluorescence studies also indicated elevated levels of HABP1 in HA treated liver ECs as compared to untreated ones (Fig. 6a). During the course of our subcellular localization studies, β- catenin was detected at the cellular periphery in the untreated murine ECs. On the contrary, HA treatment of ECs led to internalization of β- catenin to the nucleus as it was observed mostly to be co-localized with Hoechst stain, confirming nuclear localization (Fig. 6b).

## Discussion

Serum HA levels reportedly increase during chronic liver diseases together with the development of liver fibrosis with different aetiologies including Hepatis B virus (HBV), Hepatitis C virus (HCV) infection or alcoholic damage [46]. Initially, enhancement of HA production by the activated stellate cells may contribute to the increase in serum HA levels observed in patients with chronic liver disease without cirrhosis, including primary biliary cirrhosis [47] or chronic hepatitis C [16, 48]. Later, when cirrhosis is established, reduced degradation by LSEC may result in HA accumulation [16, 47]. The development of hepatic sinusoid capillarization might be able to explain the reduced HA degradation. In spite of the fact, that several studies have described serum HA as a fibrosis marker in Chronic Hepatitic C [49–53], but still there are reservations to include HA as a non-invasive marker for diagnosis of chronic HBV patients [54–57]. It is evident from clinical data, that in patients with cirrhosis and high serum HA levels are clearly associated with clinical severity, the occurrence of complications or death [58–60]. Serum HA levels are also shown to be related to the effect of interferon-α therapy on liver fibrosis [16, 61]. Therefore, it is important to understand the influence of HA in the cellular components of liver especially liver ECs.

**Fig. 7:**
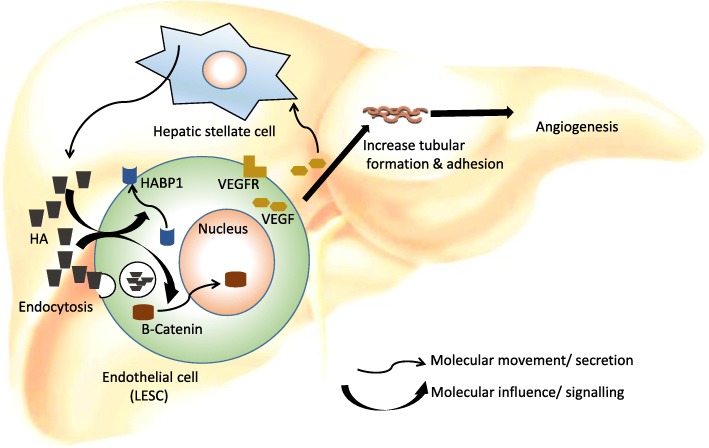
Schematic diagram showing influence of HA on angiogenesis in liver ECs. In the liver microenvironment the sinusoidal pericyte, hepatic stellate cells (HSCs) synthesize HA and this compound degraded by the sinusoidal ECs via the process of endocytosis [16]. Elevated HA level or administration of HA are co-ordinate with expression of HABP1 and internalization of β- catenin in ECs. HA treatment enhanced the tubular sprout formation, rearrangement of the actin cytoskeleton, cell migration and adhesion along with overexpression of angiogenic factors VEGFA and VEGFR1 in liver ECs

ECs contain unusually high amounts of endocytic vesicles, which suggests their engagement in uptake of proteins from blood passing through the sinusoids [26]. Most interestingly the first physiological macromolecule shown to be cleared from the circulation by ECs is HA [62, 63]. Co-ordinated functional role of HA and HABP1 in liver ECs in term of angiogenesis tempted us to understand the therapeutic implications of ECs. Previous reports elucidated the fact, that overexpression of HABP1 in the human liver cell line HepG2, induces higher endogenous HA level and inter-cellular HA cables, glutathione levels and enhanced cellular proliferation over longer periods of cell growth. Enhanced levels of hyaluronan synthase 2 (HAS2) and CD44, a receptor of HA has also been reported in the same study. The elevated level of HABP1 thus, leads to enhanced tumorigenic potential of the hepatocarcinoma cells, by HA mediated pathways with upregulation of AKT, *p-*AKT, β-catenin and cyclinD1 [10]. The cell surface expressed form of HABP1 has been predicted as a new biomarker of tumor cells and tumor associated macrophages in metabolically deprived or hypoxic areas of tumor [64]. In the present study, our findings have suggested exogenous administration of HA accompanied by elevated levels of HABP1 and complete dispersion throughout the cell might have a crucial impact in inducing cellular proliferation and enhanced adhesion as well as cell migration in ECs. The diverse subcellular localization of HABP1 upon HA treatment suggest that it could be a component of the trafficking pathway connecting the nucleus, mitochondria and cytoplasm and the export pathway to the cell surface (Fig. 7). Upon examining the pro-angiogenic action of HA and and its effect on angiogenesis, it has been revealed that even HA supplementation in the media is sufficient enough to promote vessel formation and confer a pro-angiogenic phenotype to endothelial cells in vitro. Very recent studies have interpreted the crucial events on interactions of HA with its cellular receptor CD44 regulating the progression of tumor [65, 66]. In fact matrix HA endorses specific microRNA, which leads to drug resistance apart from influencing tumor progression. MicroRNAs reportedly regulate the post-translational modifications of HA synthases (HASes) which impacts the HA synthesis and secretion in the tumor microenvironment [65, 67].

In this context, VEGF and its receptor, VEGFR1 are most vital factors during angiogenesis, having a central role in EC proliferation. Our work confirmed the overexpression of these potent angiogenic factors, VEGFA and VEGFR1 upon HA treatment both at the transcript and protein levels [2, 3]. Here, we confirmed the effect of soluble HA upon the rearrangement of the actin cytoskeletal. Thus, HA-mediated signalling pathways are likely to regulate actin cytoskeletal organization and cell migration. Among multiple pathways that have been reported to participate in the regulation of tumor angiogenesis and metastasis, Wnt/β-catenin signalling pathways contribute to hepatocellular carcinoma. Increased levels of HA are reported to induce cell survival pathway via the activation of AKT and β- catenin [10, 68]. On the other hand, β-catenin pathway has also been reported to regulate the expression of VEGF in colorectal cancer [34]. Canonical Wnt/β-catenin signalling regulates gene transcription by enabling translocation of β-catenin from the cytoplasm into the nucleus [33]. In our experimental system, we not only observed upregulation of β-catenin expression, but also detected the nuclear translocation of the protein from the membrane upon HA treatment (Fig. 7). Furthermore, nuclear factor, NF-κB is pivotal in the regulation of many genes including adhesion molecules. Recent studies imply that, phosphorylation and degradation of IκB is not enough for NF-κB dependent transcription. Phosphorylation of p65 subunit of NF-κB or RelA can also activate NF-κB-dependent transcription [69, 70]. In fact, earlier studies from the lab revealed HABP1 induced migration of melanoma cells, as well as tumor growth by NF-κB dependent MMP-2 activation [69]. Since HA treated ECs have been found to be more adhesive in nature, we analysed the transcript levels of p65 subunit of NF-κB along with the NF-κB1 (p50 subunit). Although HA treatment did not influence the mRNA levels of NF-kB1, but a highly elevated mRNA level of p65 subunit of NF-κB has been elucidated from the study. This observation relegates a critical question for future studies to determine how these pathways interact to facilitate angiogenesis.

## Conclusions

Our study has shed light on five major piece of evidence; HA mediated signaling in liver ECs increases i) proliferation, migration and adhesion ii) tubular formation and alteration in cytoskeletal organization iii) expression of pro-angiogenic factors iv) dispersion and recruitment of HABP1 towards cellular membrane to influence HA mediated signaling v) mobilization of β-catenin to nucleus for transcriptional activation of angiogenesis. The elucidation of the molecular mechanisms regulating HA-mediated cellular signalling in ECs will not only contribute greatly to our understanding of cancer progression but will also offer many new avenues of therapeutic intervention. ECs should be considered as an attractive model for future and existing anti-angiogenic strategies directed against the stromal compartment of liver metastases.

## Additional file

Additional file 1: **Table S1.** Sequences of primers used for quantitative real time PCR on mice. (DOCX 25 kb)

## Abbreviations

EC: Endothelial cells
GAPDH: Glyceraldehyde-3-phosphate dehydrogenase
HA: Hyaluronic acid or Hyaluronan
HABP1: Hyaluronic acid binding protein1
NF-κB: Nuclear factor kappa-light-chain-enhancer of activated B
RHAMM: Receptor for hyaluronan mediated motility
VECAM: Vascular endothelial cell adhesion molecule
VEGFA: Vascular endothelial growth factor A
VEGFR1: Vascular endothelial growth factor receptor 1
