# Supplementary material for: Dynamic Hyaluronan drives liver endothelial cells towards angiogenesis
Source: BMC Cancer. 2018 Jun 11;18:648. doi: 10.1186/s12885-018-4532-1 (PMC5996548; doi:10.1186/s12885-018-4532-1)
Supplement: Supplementary file 1 — Table S1. Sequences of primers used for quantitative real time PCR on mice. (DOCX 25 kb) [file 12885_2018_4532_MOESM1_ESM.docx]

Aditional file

Additional file 1 Table S1. Sequences of primers used for quantitative real time PCR on mice

| **Gene** | **Forward primers (5’to 3’)** | **Reverse primers (5’to 3’)** |
| --- | --- | --- |
| VEGFA | CAGGCTGCTGTAACGATGAA | TTTCTTGCGCTTTCGTTTTT |
| VEGFR1 | ACCTCCGTGCATGTGTATGA | TGGTGCATGGTTCTGTTGTT |
| HABP 1 | ATCAACTCCCAATTTCGTGGTT | TCCTCTGGATAATGACAGTCCAA |
| RHAMM | TACAGAGGCAGCATTCCCAG | TGTAGTTCAATGACATCATAGCCA |
| β-Catenin | GTGCTATCTGTCTGCTCTAGTA | CTTCCTGTTTAGTTGCAGCATC |
| VECAM | CCCAGGTGGAGGTCTACTCA | CAGGATTTTGGGAGCTGGTA |
| NF-kB1 | GAAATTCCTGATCCAGACAAAAAC | ATCACTTCAATGGCCTCTGTGTAG |
| p65, NF-kB | CTTCCTCAGCCATGGTACCTCT | CAAGTCTTCATCAGCATCAAACTG |
| GAPDH | AGGTCGGTGTGAACGGATTTG | TGTAGACCATGTAGTTGAGGTCA |
